# Supplementary material for: Efficacy of Combining an Extraoral High-Volume Evacuator with Preprocedural Mouth Rinsing in Reducing Aerosol Contamination Produced by Ultrasonic Scaling
Source: Int J Environ Res Public Health. 2022 May 16;19(10):6048. doi: 10.3390/ijerph19106048 (PMC9141298; doi:10.3390/ijerph19106048)
Supplement: Supplementary file 1 [file ijerph-19-06048-s001.zip › ijerph-1712461-supplementary.pdf]

Supplemental data

CFUs (20 cm)

	DM	DC	PC	AA	BT
No-rinsing	14.7±8.3	32.6±21.6	180.9±18.7	3.2±3.6	2.1±2.1
DW	5.1±5.2	12.4±15.4	136.2±29.3	1.0±1.5	1.4±1.6
PI	6.9±8.9	6.6±5.4	59.8±27.9	2.4±3.6	0.6±1.1
EO	5.7±5.2	13.4±13.8	68.6±32.2	2.3±2.8	1.3±1.6

CFUs (10 cm)

	DM	DC	PC	AA	BT
No-rinsing	1.5±1.0	2.2±2.0	20.3±10.0	0.9±1.3	1.8±1.5
DW	1.0±0.8	1.5±1.0	10.0±5.0	0.9±0.5	1.0±0.7
PI	0.7±0.5	0.7±0.5	6.0±5.0	0.9±0.5	0.9±0.7
EO	1.0±0.4	1.0±0.4	7.0±4.0	0.9±0.4	0.9±0.6

RLUs (20 cm)

	DM	DC	PC	AA	BT
No-rinsing	41.0±18.9	30.8±9.4	408.7±127	31.4±14.8	30.7±9.7
DW	42.4±14.8	26.6±5.8	293.1±127	39.6±22	33.7±16.0
PI	24.2±16.7	18±13.9	88.4±54.5	10.5±4.9	18.5±3.5
EO	20.7±17.7	23.2±14.8	126.8±90.5	27.3±8.0	38.4±10.7

RLUs (10 cm)

	DM	DC	PC	AA	BT
No-rinsing	14.2±5.0	4.9±4.0	21.4±10.0	11.0±8.0	2.5±2.0
DW	10.0±10.0	4.0±4.0	20.0±5.0	10.0±7.0	2.0±2.0
PI	10.0±7.0	5.0±3.0	10.0±7.0	10.0±7.0	2.0±2.0
EO	10.0±7.0	5.0±3.0	10.0±7.0	10.0±7.0	2.0±2.0

The results of CFU counts and ATP assay are shown in Table. The values in the parentheses are the distances between eHVE and the mouth.

DM: doctor's mask; DC: doctor's chest area; PC: patient's chest area; AA: assistant's area; BT: bracket table; NR: no rinsing; DW: distilled water; PI: povidone iodine; EO: essential oil.
